# Integrative Taxonomy Reshapes Palaearctic–Oriental Biogeography: First Discovery of *Dicranomyia* (*Sivalimnobia*) (Diptera, Limoniidae) in Mainland China with Two New Species †

**DOI:** 10.3390/insects17070690

**Published:** 2026-07-02

**Authors:** Liying Dai, Pengxuan Guo, Xiao Zhang

**Affiliations:** Shandong Engineering Research Center for Environment-Friendly Agricultural Pest Management, College of Plant Health and Medicine, Qingdao Agricultural University, Qingdao 266109, China; dailiying29@163.com (L.D.); gpx183213@163.com (P.G.)

**Keywords:** Chinese fauna, new species, new record, integrative taxonomy, DNA barcoding, biogeography

## Abstract

The crane fly subgenus *Sivalimnobia* has been taxonomically stagnant for over half a century, with its apparent absence from mainland China representing a major biogeographic anomaly. We address this by integrating detailed morphology and COI DNA barcoding of specimens collected across 14 Chinese provinces. Our study conclusively establishes the presence of *Sivalimnobia* in mainland China, resolving three species: the newly recorded and continentally widespread *Dicranomyia* (*Sivalimnobia*) *alticola* Edwards, 1916, and two new endemic species—*D*. (*S*.) *bispinosa* sp. nov. (restricted to Yunnan) and *D*. (*S*.) *inflata* sp. nov. (a cryptic lineage within the *D*. (*S*.) *alticola* complex distributed across Chongqing, Sichuan and Yunnan). The discovery of *D*. (*S*.) *inflata* sp. nov. was validated by a discrete male genitalic autapomorphy and a pronounced COI barcode gap (8.7–9.6% divergence). These findings update the global biogeography of the subgenus, identifying India as the primary diversity center and southwestern China as a significant secondary center. The overall pattern suggests a “stepping-stone dispersal and multi-center diversification” model. This work transforms *Sivalimnobia* from a taxonomic relic into a model for studying Eurasian insect biogeography and highlights the conservation importance of East Asian montane stream habitats.

## Simple Summary

For over 50 years, the classification of a group of moisture-loving crane flies, *Dicranomyia* (*Sivalimnobia*), had seen no progress, and these insects were curiously missing from mainland China—a region seemingly ideal for them. To solve this puzzle, we collected and studied these flies across China using both physical examination and analyzing their DNA. We confirmed their presence in mainland China for the first time, discovering three distinct kinds: one widespread, known species now found across much of the country, and two completely new species that live only in small, isolated areas of the southwestern mountains. Our findings redraw the global map for this group, showing that India is a main hotspot, and southwestern China is a new, important second center of diversity. This study brings a forgotten insect group back into the spotlight, making it a useful example for understanding how species evolve and spread across Asia, and emphasizes the need to protect the unique mountain stream habitats they call home.

## 1. Introduction

The crane fly genus *Dicranomyia* Stephens, 1829 represents one of the most species-rich yet taxonomically intricate lineages in the family Limoniidae (Diptera), comprising over 1100 morphologically diverse species distributed across all biogeographic realms [1]. However, this remarkable diversity, coupled with frequently subtle interspecific differences and a historical reliance on variable characters such as wing venation, has rendered *Dicranomyia* notoriously challenging to classify, leaving many of its species-groups and subgenera in need of critical revision. Among the 24 currently recognized subgenera of *Dicranomyia*, *Sivalimnobia* Alexander, 1963 stands out due to its distinctive and stable morphology. Alexander established this subgenus to accommodate a peculiar and phylogenetically isolated species group, defining it not merely on the relative length of vein Sc, but on consistent and complex genitalic structures in both sexes in 1963 [2]. Specifically, the male hypopygium possesses a uniquely modified inner gonostylus bearing a characteristic rostral arm and a separate basal spinous arm, while the female ovipositor exhibits greatly reduced cercus and correspondingly enlarged hypogynial valva. Ecologically, species of *Sivalimnobia* are specialized inhabitants of persistently humid riparian microhabitats—including waterfalls, vertical seepages and forested stream splash zones—as documented in *D*. (*S*.) *aquosa* Verrall, 1886 and *D*. (*S*.) *euphileta* (Alexander, 1924) [3,4,5].

Despite its clear morphological and ecological definition, *Sivalimnobia* has remained in a state of profound taxonomic stasis for over half a century. Following the description of the first species by Verrall in 1886 [6], an active study period from 1912 to 1973 saw the description of the remaining 14 species [2,7,8,9,10,11,12,13,14,15,16,17], completing the 15 currently recognized species. Distributed across the Palaearctic and Oriental regions, their diversity center is hypothesized to be in India [2]. Since 1973, no new species have been published, and records have been limited to sporadic mentions in regional faunal surveys (e.g., Podenas et al. 2019 for Korea [4]; Kato 2023, 2024 for Japan [18,19]), effectively rendering this subgenus a taxonomic “time capsule” of mid-20th-century entomology. Its prolonged stasis is further accentuated by a conspicuous biogeographic anomaly: the complete absence of *Sivalimnobia* from mainland China. Its known range extends from Western Europe (e.g., the United Kingdom and France) through the Russian Far East, Japan and Korea, southward to Southeast Asia (Malaysia, Indonesia, and Philippines) and westward to India, with only a single historical record from Taiwan, China [1]. Given the vast and ecologically varied terrain of mainland China, particularly its extensive Oriental region rich in mountainous stream systems ostensibly ideal for such hygrophilous taxa, this distributional gap appears not only glaring but logically untenable. It strongly suggests that the current 15 species substantially underestimate the subgenus’s actual diversity, and that the gap results more from sampling bias than true biogeographic absence.

Consequently, this prolonged taxonomic stasis, juxtaposed with a glaring biogeographic gap, gives rise to a series of interlinked questions. First, is the apparent species poverty of *Sivalimnobia* real, or merely an artifact of insufficient sampling in its specialized habitats? If sampling bias is the primary cause—as the Chinese distributional void strongly implies—then what levels of hidden diversity and novel distribution patterns remain undocumented, particularly across the vast and under-surveyed terrain of mainland China? To resolve these uncertainties and break the five-decade silence surrounding this group, we initiated a targeted survey across mainland China. Employing an integrative taxonomic framework, our study aimed to definitively establish the presence or absence of *Sivalimnobia* in the region, assess its diversity and delineate species boundaries by coupling detailed morphology with COI DNA barcoding, and thereby revise the subgenus’s distributional framework. Here we confirm, for the first time, the widespread presence of *Sivalimnobia* in mainland China. This discovery, which includes one widely distributed species and two new species, not only closes a major biogeographic gap but also fundamentally reshapes our understanding of this long-neglected lineage.

## 2. Materials and Methods

### 2.1. Specimen Collection and Preservation

The specimens used in this study were primarily obtained from the collections of our institution and its partner institutions. On this basis, additional surveys were conducted using net sweeping, light trapping and Malaise trapping methods. The specimens were collected from 2003 to 2019, covering 14 provinces in China, with a total of over 170 specimens obtained. The specimens were immediately placed in vials, with labels containing detailed information including geographical location, collection date, and, where applicable, altitude, and were then placed in 95% ethanol (Fuyu Fine Chemical, Tianjin, China) for long-term preservation at –20 °C in the collection repository of Qingdao Agricultural University, ensuring morphological integrity for subsequent morphological studies.

### 2.2. Morphological Observation and Documentation

Morphological identification was primarily performed using a Phenix SMZ180-LB stereomicroscope (Phenix Optics, Shangrao, Jiangxi, China), with the specimens submerged in 95% ethanol during observation to enhance the clarity of morphological features. For genital structures requiring detailed examination through dissection, the specimens were immersed in a cold 10% sodium hydroxide (NaOH) (Aladdin, Shanghai, China) solution for 12 to 15 h to sufficiently soften tissues and remove adherent matter, thereby allowing clear observation of internal structures. Photography was conducted using Canon EOS 5D (Canon, Tokyo, Japan) and 90D digital cameras (Canon, Tokyo, Japan) equipped with macro lenses for high-precision imaging, during which the specimens were placed in 95% ethanol or glycerin (Debao Biotech, Guangzhou, Guangdong, China) to enhance the rendering of fine details and ensure that diagnostic characters were clearly discernible. The morphological terminology in this study primarily follows Cumming and Wood [20], while the interpretation of wing venation adheres to the system of de Jong [21].

### 2.3. DNA Extraction, PCR Amplification, and Sequencing

In this study, six specimens of *Sivalimnobia* were successfully sequenced (detailed information is provided in Table 1). Genomic DNA was extracted from thoracic muscle tissue using the TIANamp Genomic DNA Kit (TIANGEN, Beijing, China), and all the procedures were performed following the manufacturer’s instructions. The mitochondrial cytochrome c oxidase subunit I (COI) barcode region was amplified via polymerase chain reaction (PCR) using the universal primers LCO1490 (5′-GGGTCAACAAATCATAAAGATATTGG-3′) and HCO2198 (5′-TAAACTTCAGGGTGACCAAAAAATCA-3′) [22]. The PCR mixture had a total volume of 25.0 μL, consisting of 12.5 μL of Taq PCR Master Mix, 1.0 μL of genomic DNA template, 1.0 μL of each primer and 9.5 μL of double-distilled water. The amplification program was performed under the following thermal cycling conditions: initial denaturation at 94 °C for 4 min; followed by 30 cycles of denaturation at 94 °C for 30 s, annealing at 46 °C for 35 s, and extension at 72 °C for 1 min; with a final extension at 72 °C for 10 min. Purification of the amplified products and subsequent Sanger sequencing were completed by Sangon Biotech (Shanghai, China).

### 2.4. Phylogenetic and Genetic Distance Analyses

The phylogenetic reconstruction incorporated a total of eight COI sequences. Among these, six sequences representing three Chinese species were generated for the first time in this study, whereas the remaining two sequences were obtained from the Barcode of Life Data System (BOLD) database [23], namely *D*. (*D*.) *brevivena* Osten Sacken, 1869 (process ID: ABOTH9532-23) and *D*. (*D*.) *chillcotti* (Alexander, 1968) (process ID: ABOTH8936-22). Multiple sequence alignments were conducted based on codon positions using the ClustalW algorithm, as implemented in MEGA 7 [24]. Phylogenetic relationships were inferred employing the maximum likelihood (ML) optimality criterion, with the Kimura 2-parameter (K2P) substitution model selected in MEGA 7. Nodal support was evaluated through 1000 bootstrap pseudoreplicates. Interspecific and intraspecific genetic distances among the subgenus *Sivalimnobia* sequences were also computed under the K2P model using MEGA 12 [25].

## 3. Results

### 3.1. Molecular Analysis

**Figure 1:**
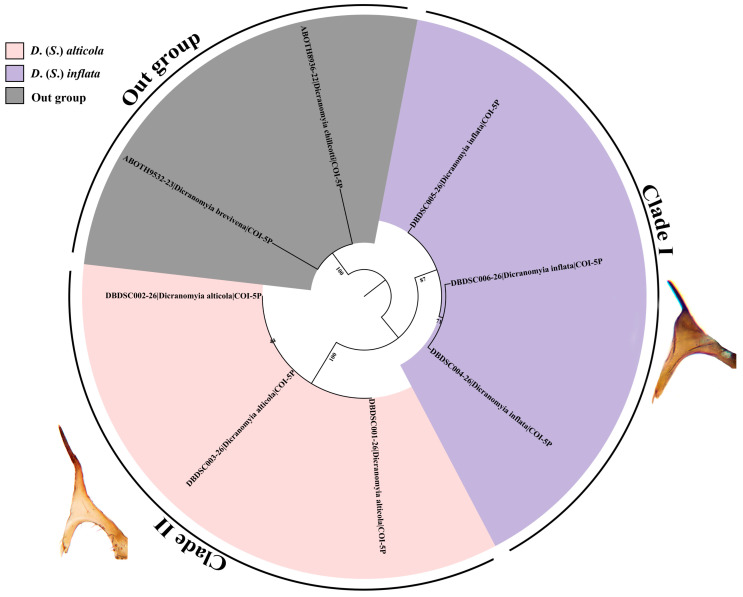
Phylogenetic tree of the subgenus *Sivalimnobia* based on COI gene barcoding, showing a clear separation into two clades. Adjacent to each of the two clades is a photograph illustrating the dorsal prolongation of the inner gonostylus of the respective species.

Morphological examination of *Sivalimnobia* specimens from 14 Chinese provinces revealed two distinct patterns. One specimen from Yunnan exhibited significant and consistent divergence in genitalic structures from all the known species, warranting its recognition as a new species (*D*. (*S*.) *bispinosa* sp. nov.; see taxonomic description below). The remaining specimens largely corresponded to the known species *D*. (*S*.) *alticola* Edwards, 1916 from Taiwan, China, though while continuous macro morphological variation was noted across populations, a discrete and stable character—a distinct swelling of the dorsal prolongation of the male hypopygial inner gonostylus—was consistently observed in three populations (two from Sichuan and one from Yunnan), sharply contrasting with typical *alticola*. To explore the taxonomic significance of this morphological variation, we selected representative samples from the three populations with enlarged dorsal prolongation of the male hypopygial and three typical populations of *D*. (*S*.) *alticola* for COI gene barcoding and phylogenetic analysis. The resulting phylogenetic tree (Figure 1), constructed using the maximum likelihood method, clearly separated all the samples into two clades. One clade (Clade I) included the three populations with enlarged ventral dorsal prolongation, while the other clade (Clade II) consisted of the typical *D*. (*S*.) *alticola* populations. This phylogenetic separation demonstrated perfect concordance with the observed morphological dichotomy, suggesting the morphological character represents a genuine evolutionary divergence rather than phenotypic plasticity or population-level variation.

Genetic distance analysis (Table 2) provided quantitative support for the phylogenetic and morphological distinctions observed between the two clades. The average genetic distance between Clade I and Clade II was found to be 8.7–9.6%, which is well beyond the typical intraspecific variation threshold (2–3% in insects) and indicates a level of divergence comparable to that of distinct species. Within each clade, however, the genetic distances were low, with values of 1.5–2.2% for Clade I and 0–0.2% for Clade II. The pronounced “barcode gap”—characterized by the clear discontinuity between low within-clade and high between-clade genetic distances—provides robust molecular evidence for recognizing Clade I as an independent evolutionary lineage. Consequently, the integrated morphological and molecular data strongly support the taxonomic distinction of the populations comprising Clade I as a previously unrecognized cryptic species, which we formally designate as *D*. (*S*.) *inflata* sp. nov. in the subsequent taxonomic description.

### 3.2. Taxonomic Treatment

This revision synthesizes the morphological analysis of newly collected *Sivalimnobia* specimens from China with thorough examination of the published literature for all previously known species to ensure accurate species delimitation. This section provides full descriptions of the *Sivalimnobia* species currently confirmed from China, two of which are new to science, along with a diagnostic key for the three species.

#### 3.2.1. *Dicranomyia* (*Sivalimnobia*) *alticola* Edwards, 1916

*Dicranomyia alticola* Edwards, 1916. Ann. Mag. Nat. Hist. (8) 18: 246. Type locality: China: Taiwan, Horisha, Arisan.

Material examined. 1 male, China: Fujian Province, Wuyishan City, Miaowan, 27 April 2009, Tingting Zhang. 1 male, China: Fujian Province, Wuyishan City, 5 August 2019, Li Shi and Xiaoyan Liu. 1 male, China: Fujian Province, Nanping City, Wuyishan National Park, Dazhulan (975 m), 3 July 2009, Li Shi and Xiaoyan Liu. 5 females, 36 males, China: Guangdong Province, Ruyuan County, Nanling Mountains, 24 March 2003, Ding Yang. 1 male, China: Guizhou Province, Suiyang County, Kuankuoshui Central Station, 11 July 2010, Sipei Liu. 2 males, China: Guizhou Province, Suiyang County, Kuankuoshui Central Station, 10 August 2010, Sipei Liu. 3 males, China: Guizhou Province, Suiyang County, Kuankuoshui Central Station, 11 August 2010, Sipei Liu. 2 males, China: Inner Mongolia Autonomous Region, Dongsheng District, 7 August 2006, Maoling Sheng. 1 male, China: Hubei Province, Shennongjia Forestry District, Guanmenshan Protection Station, 18 July 2007, Qifei Liu. 1 male, China: Hunan Province, Sangzhi County, Badagong Mountain Township (1000 m), 22 July 2012, Mingchao Huang. 2 females, 4 males, China: Hunan Province, Sangzhi County, Tianping Mountain (1300 m), 25 July 2012. 1 male, China: Hunan Province, Sangzhi County, Tianping Mountain (1300 m), 24 July 2012; Mingchao Huang. 1 male, China: Hunan Province, Taoyuan County, Zhushan Village, 11 September 2016, Liang Wang. 5 females, 15 males, China: Hunan Province, Zhangjiajie City, Sangzhi County, Tianping Mountain (1500 m), 18 June 2014, Xiao Zhang. 2 males, China: Jiangxi Province, Jinggangshan City, Ciping District Fengshuping, 1 August 2014, Kai Wang. 1 female, 2 males, China: Jiangxi Province, Jinggangshan City, Jinggang Mountain Zhusha Forest Farm (600 m), 9 October 2012. 1 male, China: Shaanxi Province, Xian City Zhouzhicheng Village, Old Town Site (2057 m), 20 August 2014, Xuankun Li. 2 males, China: Sichuan Province, Emeishan City, Mount Emei, Qingyin Pavilion, 1 July 2010, Tao Li. 2 males, China: Sichuan Province, Pingwu County, Laohegou Nature Reserve, Heigou, 14 May 2016, Zehui Kang. 1 male, China: Sichuan Province, Muli Xizang Autonomous County, 24–26 July 2019, Liang Wang. 1 male, China: Taiwan Province, Chiayi County, Alishan Township, Lijia Village (1500 m), 6 June 2012, Hui Dong. 1 male, China: Xizang Autonomous Region, Bomi County (2700 m), 12–26 July 2016, Shaolin Han (M). 2 females, 6 males, China: Yunnan Province, Lvchun County, Yakou Reservoir (1779 m), 26 February 2019, Liang Wang. 9 males, China: Yunnan Province, Lvchun County, Yakou Reservoir (1779 m), 26 March 2019, Liang Wang. 2 females, 3 males, China: Yunnan Province, Lvchun County, Qimaba Township, 28 March 2019, Liang Wang. 1 male, China: Yunnan Province, Lvchun County, Qimaba Township (1378 m), 28 March 2019, Xin Li. 4 females, 11 males, China: Zhejiang Province, Suichang County, Jiulong Mountain, Xikengli Protection Station, 25 July 2019, Xingyang Qian.

Diagnosis. Prescutum and presutural scutum varied from yellow to dark brown, with three broad longitudinal stripes faintly visible in darker individuals. Postsutural scutum varied from yellow to pale brown, with pale areas medially and laterally when it is dark. Femora brownish yellow, with a broad dark apical ring. Pleuron yellow, darker near base of wing. Vein Sc ending opposite about 2/5–2/3 of Rs; sc-r varies from very close to tip of Sc to far beyond it (up to 3.0 times its own length). Dorsal prolongation of inner gonostylus T-shaped, upper arm slender, curved at subtip, with an apical spine about half length of upper arm. Paramere finger-shaped, tip acute, with extreme tip bending inward abruptly. Aedeagus widened at base, tip forked and recurved, subtip slightly constricted.

Description. Male. Body length 7.6–7.7 mm, wing length 6.0–8.0 mm.

**Figure 2:**
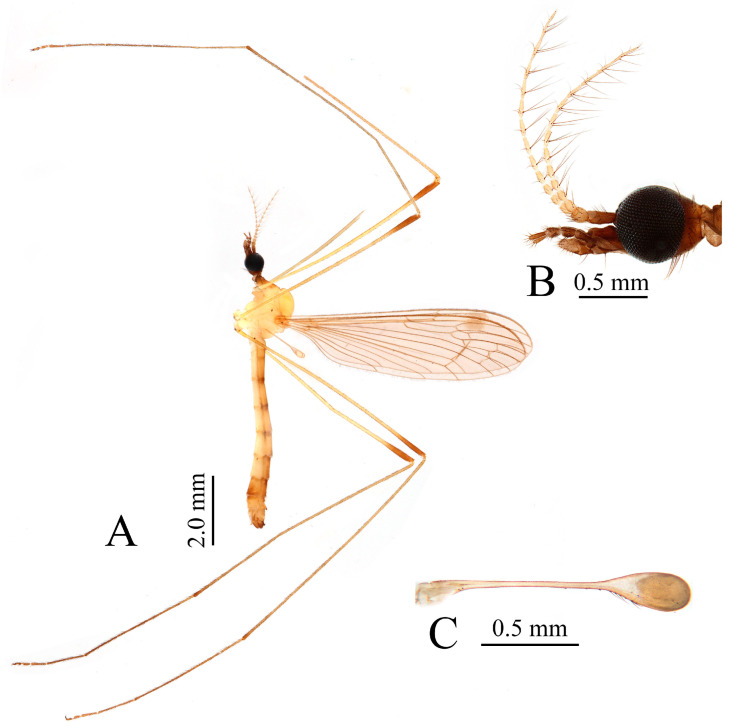
*Dicranomyia* (*Sivalimnobia*) *alticola* Edwards, 1916: (**A**) habitus of male, lateral view; (**B**) head, lateral view; (**C**) halter.

Head (Figure 2A,B). Dark brown. Setae on head brown. Antenna with 14 segments, brownish yellow with scape and pedicel darker. Scape cylindrical, 3.0 times as long as wide. Pedicel oval, length and width subequal, tip slightly enlarged. Basal three flagellomeres long-oval, remaining flagellomeres elongated, tapering apically; each flagellomere with 3–5 brown verticils, disposed in an irregular whorl, and longest exceeding length of corresponding flagellomere, outermost flagellomere with two short apical setae. Rostrum dark brown with dark brown setae. Palpus dark brown with brown setae.

**Figure 3:**
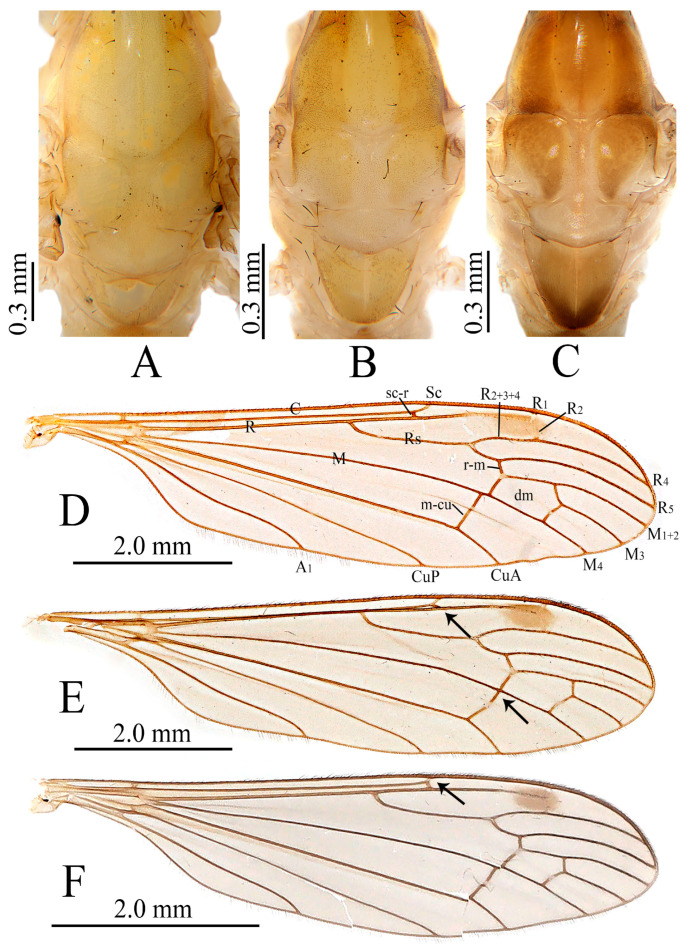
*Dicranomyia* (*Sivalimnobia*) *alticola* Edwards, 1916: (**A**–**C**) variations in thorax, dorsal view; (**D**–**F**) variations in wing. The black arrows in (**E**) indicate intraspecific variation in the position of sc-r and m-cu, while that in (**F**) indicates variation in the termination point of Sc; these features are not taxonomically diagnostic within *D*. (*S*.) *alticola*.

Thorax (Figure 2A and Figure 3A–C). Cervical sclerite dark brown. Pronotum brownish yellow, with anterior area darker. Prescutum and presutural scutum varied from yellow to dark brown (Figure 3A–C), with three broad longitudinal stripes faintly visible in darker individuals (Figure 3C). Postsutural scutum varied from yellow to pale brown (Figure 3A–C), with pale areas medially and laterally when it is dark (Figure 3C). Scutellum yellow (Figure 3A–C). Mediotergite varied from yellow to brown (Figure 3A–C), with a darker area on posterior margin when it is dark (Figure 3C). Pleuron yellow, darker near base of wing (Figure 2A). Thorax with dark brown setae, densely covered with fine brown pubescence (Figure 3A–C). Coxae (Figure 2A) pale yellow; trochanters slightly darker in comparison to coxae; femora brownish yellow, with a broad dark apical ring; tibiae pale brown with tips slightly darker; tarsi pale brown. Setae on legs brown. Wing brownish yellow to pale brown, stigma distinctly long-oval and darker; veins brown. Venation: Sc ending opposite about 2/5–2/3 of Rs (Figure 3D–F); sc-r varies from very close to tip of Sc to far beyond it (up to 3.0 times its own length); Rs arising from mid-wing, base slightly curved; cell dm closed, 1.5–2.0 times as long as wide; m-cu before (Figure 3D) or at (Figure 3E) base of dm. Halter (Figure 2C) brownish yellow, knob darker.

Abdomen (Figure 2A). Segments 1–6 pale brownish yellow to pale brown, each segment darker at posterior margin, segment 7 brown; segment 8 brownish yellow. Setae on abdomen brown.

**Figure 4:**
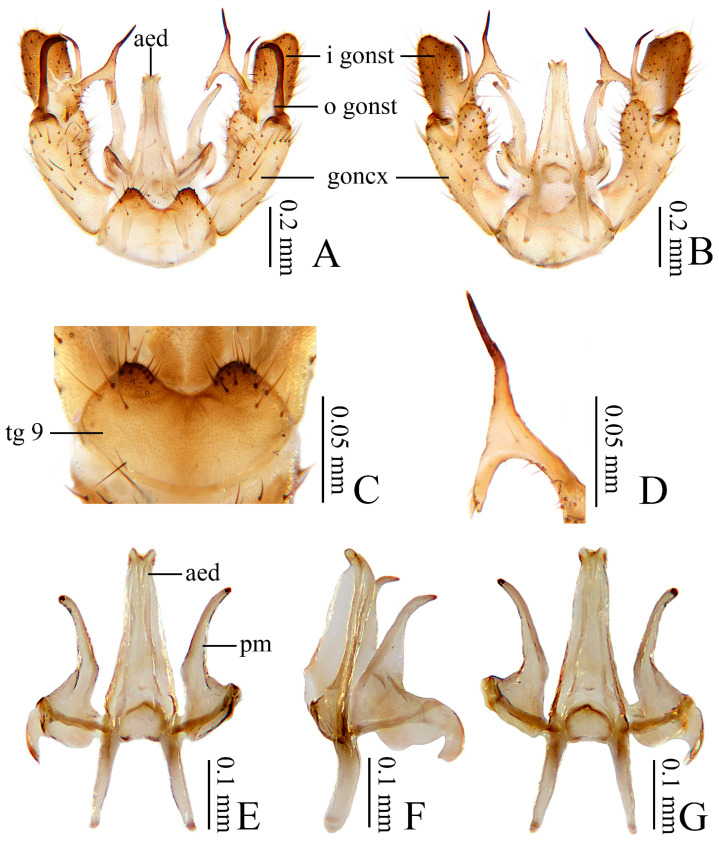
*Dicranomyia* (*Sivalimnobia*) *alticola* Edwards, 1916: (**A**) male hypopygium, dorsal view; (**B**) male hypopygium, ventral view; (**C**) tergite 9, dorsal view; (**D**) dorsal prolongation, dorsal view; (**E**) complex of aedeagus, dorsal view; (**F**) complex of aedeagus, lateral view; (**G**) complex of aedeagus, ventral view. Abbreviations employed in the illustrations are as follows: aed = aedeagus; goncx = gonocoxite; i gonst = inner gonostylus; o gonst = outer gonostylus; pm = paramere; tg = tergite.

Hypopygium (Figure 4). Generally brownish yellow to brown. Tergite 9 (Figure 4A,C) nearly trapeziform, posterior margin with a broad and distinct U-shaped notch, each lobe with about 10–15 long brown setae. Gonocoxite (Figure 4A,B) cylindrical, base and middle slightly thickened, with an elongated ventromesal lobe at middle, length about half of gonocoxite; gonocoxite with short pale brown setae, ventromesal lobe with dense short brown setae. Outer gonostylus (Figure 4A,B) sclerotized, slender, constricted at about midlength, apical third incurved, brownish black with extreme tip slightly darker. Inner gonostylus (Figure 4A,B,D) nearly as long as outer gonostylus, shallowly concave at middle of upper margin, overall dark brown except for paler medial base; inner gonostylus with two basal prolongations: dorsal prolongation (Figure 4D) T-shaped, upper arm slender, curved at subtip, with an apical spine about half length of upper arm, lower arm with 4–6 setae at subtip; ventral prolongation long, slightly curved toward inner gonostylus, with a apical spine about 1/3 length of ventral prolongation. Paramere (Figure 4E–G) finger-shaped, broad at base, narrowed and curved outward apically, tip acute, with extreme tip bending inward abruptly. Aedeagus (Figure 4E–G) long, extending to about middle of inner gonostylus, widened at base, tip forked and recurved, subtip slightly constricted.

**Figure 5:**
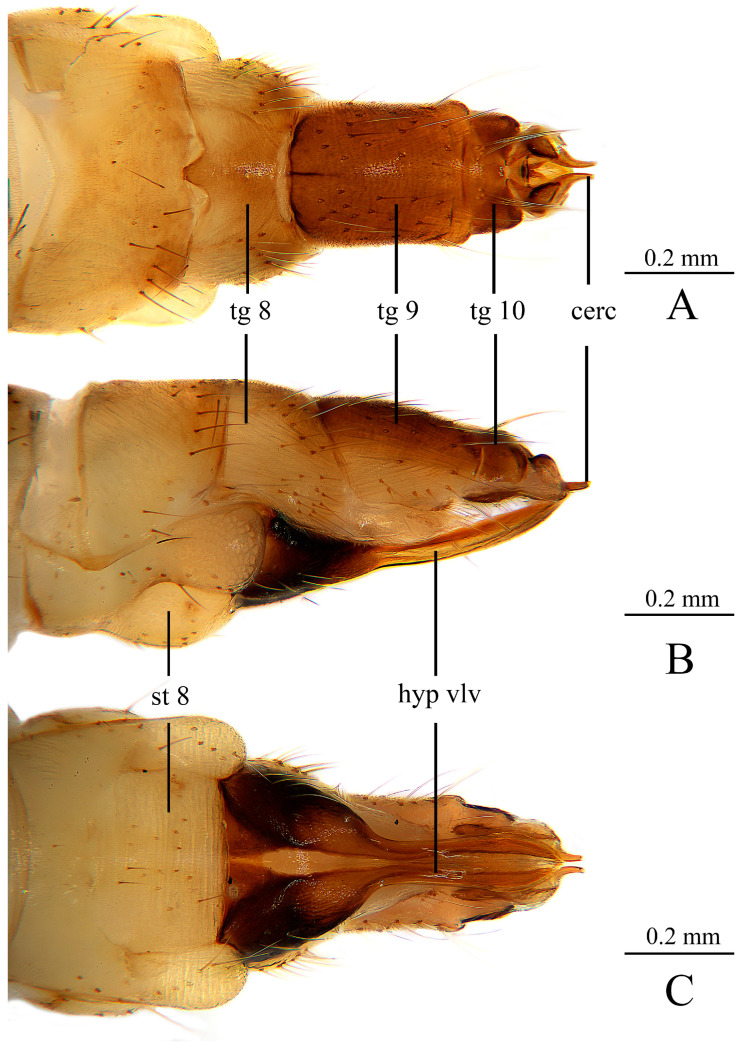
*Dicranomyia* (*Sivalimnobia*) *alticola* Edwards, 1916: (**A**) female ovipositor, dorsal view; (**B**) female ovipositor, lateral view; (**C**) female ovipositor, ventral view. Abbreviations employed in the illustrations are as follows: cerc = cercus; hyp vlv = hypogynial valve; st = sternite; tg = tergite.

**Figure 6:**
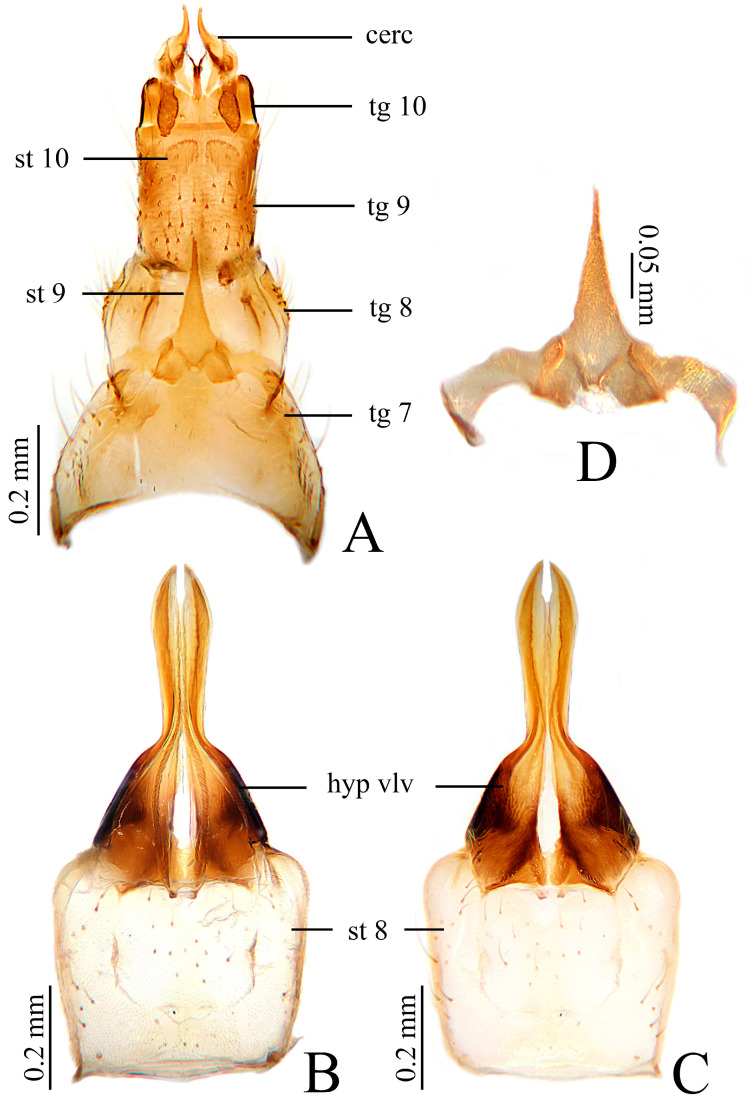
*Dicranomyia* (*Sivalimnobia*) *alticola* Edwards, 1916: (**A**) female ovipositor with sternite 8 and hypogynial valvae removed for clarity, ventral view; (**B**) sternite 8 with hypogynial valve, dorsal view; (**C**) sternite 8 with hypogynial valve, ventral view; (**D**) sternite 9, ventral view. Abbreviations employed in the illustrations are as follows: cerc = cercus; hyp vlv = hypogynial valve; st = sternite; tg = tergite.

Female. Body length 7.0–8.0 mm, wing length 7.3–8.0 mm. Generally similar to male in body coloration. Ovipositor (Figure 5 and Figure 6) with tergite 8 about 2/3 as long as tergite 9, brownish yellow, caudal margin slightly darker. Tergite 9 dark brown, caudal margin slightly paler. Tergite 10 about 1/4 as long as tergite 9, dark brown. Cercus short, apically subacute, brownish yellow. Hypogynial valve brown, with base brownish black and middle paler, extending to base of cercus. Sternite 9 (Figure 6A,D) triangular, with two quadrate lobes at base, apically tapering.

Distribution. China (Fujian, Guangdong, Guizhou, Hubei, Hunan, Inner Mongolia, Sichuan, Shaanxi, Jiangxi, Taiwan, Xizang, Yunnan, Zhejiang); Indonesia.

Remarks. This species was first described by Edwards from Taiwan in 1916 [10], China, based on two female specimens, with detailed morphological notes and a hand-drawn illustration of the female ovipositor, who also noted that the characteristics of wing vein Sc in this species blurred the taxonomic boundaries of the group within the genus “Limnobia”. Subsequently, Alexander in 1923 described the male, designating it as the synonym curvispina, and provided a detailed description of that sex [26]. Despite these previous accounts, a comprehensive modern description integrating both sexes has been lacking. Based on newly examined material, we herein provide a detailed redescription of this species, including comprehensive accounts of both male and female morphology and documentation of intraspecific variation. In terms of hypopygial characteristics, this species is similar to *D*. (*S*.) *euphileta* (Alexander, 1924), the only species of the subgenus distributed in both the Palaearctic and Oriental regions. However, the two can be distinguished by the presence or absence of darkened areas on the wing other than the stigma: in *D*. (*S*.) *euphileta*, in addition to the stigma, the costal cell in the vicinity of the cross-vein is darker [27], whereas in this species, the wing is clear except for the stigma. Regarding venation, this species also resembles *D*. (*S*.) *clavula* (Alexander 1972), described by Alexander from India, but differs in the shape of the paramere and the upper arm of the dorsal prolongation of the inner gonostylus. In *D*. (*S*.) *clavula*, the paramere has an obtuse tip, and the upper arm of the dorsal prolongation of the inner gonostylus is slightly swollen, as shown in Figure 5 of Alexander 1972 [16], whereas in this species, the paramere has an acute tip, and the upper arm of the dorsal prolongation of the inner gonostylus is slender and curved at the subtip.

#### 3.2.2. *Dicranomyia* (*Sivalimnobia*) *bispinosa* Dai & Zhang, sp. nov.

ZooBank registration number of the new species: urn:lsid:zoobank.org:act:53449B68-77AE-47DC-A1CD-63DD533AA49C.

Type material. Holotype 1 male, China: Yunnan Province, Lvchun County, Yakou Reservoir, 19 April 2018, Xin Li.

Diagnosis. Prescutum and presutural scutum dark brown, anterior area distinctly darker. Postsutural scutum brown with middle area and outer margin slightly darker. Pleuron brownish yellow, darker near base of wing. Vein Sc ending at 1/4 length of Rs; sc-r far beyond tip of Sc (up to 4.0 times its own length). Dorsal prolongation of inner gonostylus T-shaped, upper arm bearing two curved black apical spines about half length of upper arm. Paramere finger-shaped, broad at base, with extreme tip bending inward slightly. Aedeagus narrowed gradually and recurved, tip forked and bluntly rounded.

Description. Male. Body length 7.5 mm, wing length 7.8 mm.

**Figure 7:**
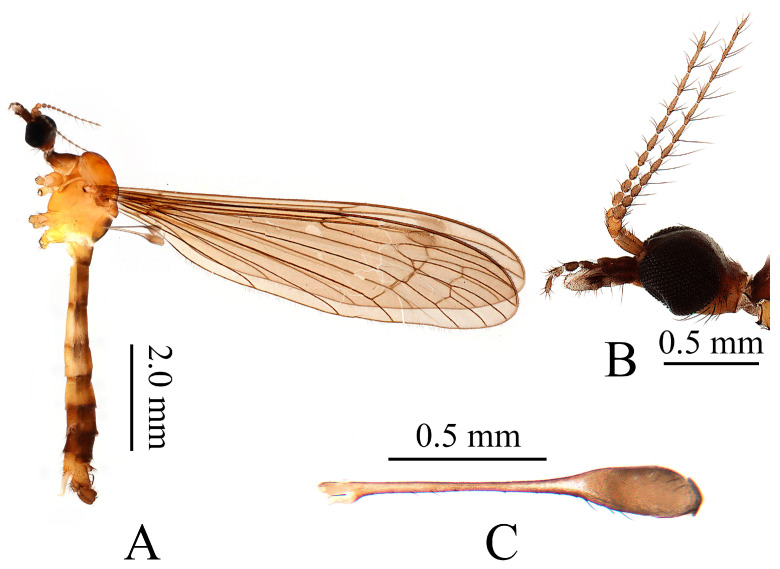
*Dicranomyia* (*Sivalimnobia*) *bispinosa* sp. nov.: (**A**) habitus of male, lateral view; (**B**) head, lateral view; (**C**) halter.

Head (Figure 7A,B). Dark brown. Setae on head brown. Antenna with 14 segments, brown, pedicel paler. Scape cylindrical, 3.0 times as long as wide. Pedicel oval. Basal two flagellomeres short-oval, remaining flagellomeres elongated, tapering apically; each flagellomere with 4–6 brown verticils, disposed in an irregular whorl, and longest exceeding length of corresponding flagellomere, outermost flagellomere with two short apical setae. Rostrum dark brown with brownish black setae. Palpus dark brown with brownish black setae.

**Figure 8:**
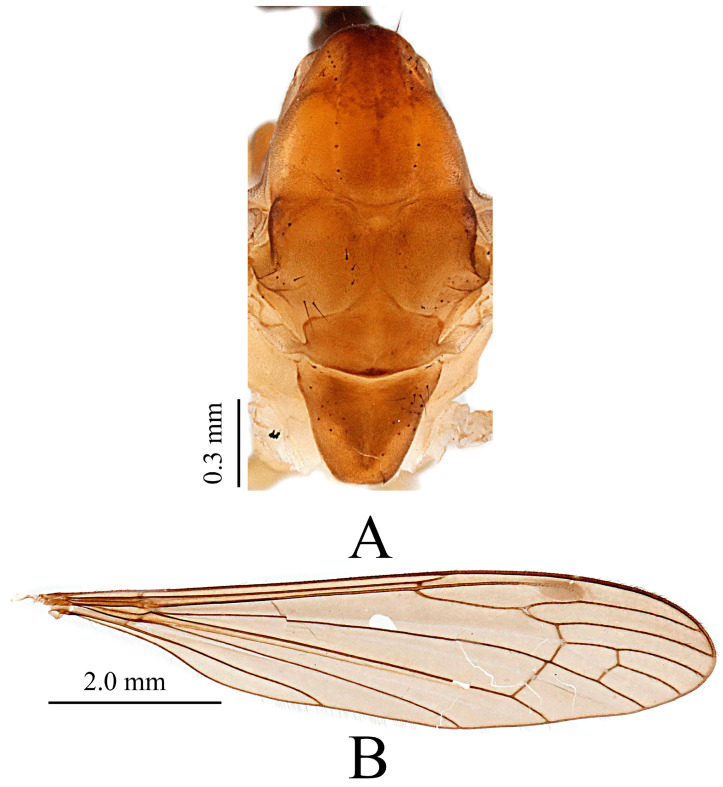
*Dicranomyia* (*Sivalimnobia*) *bispinosa* sp. nov.: (**A**) thorax, dorsal view; (**B**) wing.

Thorax (Figure 7A and Figure 8A). Cervical sclerite dark brown. Pronotum brown, with anterior area darker. Prescutum and presutural scutum dark brown, anterior area distinctly darker. Postsutural scutum brown with middle area and outer margin slightly darker. Scutellum brown. Mediotergite darker brown, a paler area on posterior margin. Pleuron (Figure 7A) brownish yellow, darker near base of wing. Thorax with dark brown setae, densely covered with fine brown pubescence. Coxae (Figure 7A) brownish yellow; trochanters slightly darker than coxae; Setae on legs dark brown. Wing (Figure 8B) pale brown, stigma distinctly long-oval and darker; veins brown; Venation: Sc ending at 1/4 length of Rs; sc-r far beyond tip of Sc (up to 4.0 times its own length). Rs arising from middle of wing, nearly straight; cell dm closed, 2.0 times as long as wide; m-cu just before base of dm. Halter (Figure 7C) brown, knob darker.

Abdomen (Figure 7A). Segments 1–5 tergites dark brown, sternites brownish yellow; segmental junctions and adjacent areas darker, segments 6–7 brownish black, segment 8 brownish yellow. Setae on abdomen dark brown.

**Figure 9:**
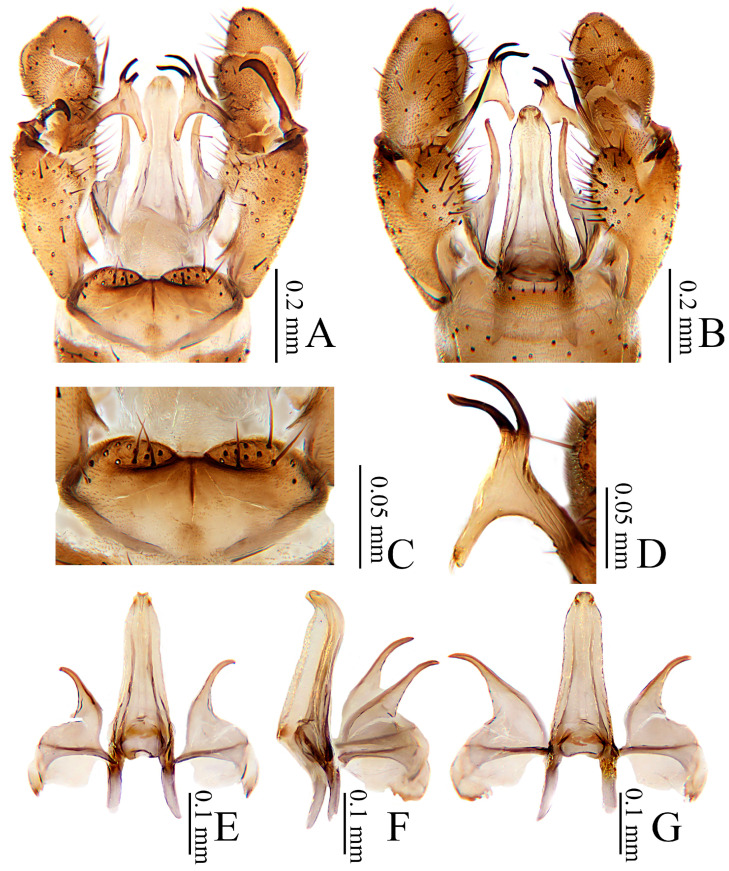
*Dicranomyia* (*Sivalimnobia*) *bispinosa* sp. nov.: (**A**) male hypopygium, dorsal view; (**B**) male hypopygium, ventral view; (**C**) tergite 9, dorsal view; (**D**) dorsal prolongation, dorsal view; (**E**) complex of aedeagus, dorsal view; (**F**) complex of aedeagus, lateral view; (**G**) complex of aedeagus, ventral view.

Hypopygium (Figure 9). Generally dark brown. Tergite 9 (Figure 9A,C) nearly trapeziform, posterior margin of tergite 9 with posterior margin medially shallowly concave, bearing a median dark brown longitudinal line, each lobe with about 8–12 long dark brown setae. Gonocoxite (Figure 9A,B) cylindrical, base contracted; with a moderate ventromesal lobe at middle, length about half that of gonocoxite; gonocoxite with dark brown setae, ventromesal lobe with dense long brown setae. Outer gonostylus (Figure 9A,B) sclerotized, expanded basally, apical 1/4 incurved and acute, overall dark brown with incurved acute portion darker. Inner gonostylus (Figure 9A,B,D) longer than outer gonostylus, shallowly concave at 1/3 of upper margin, overall brown, margin on side toward aedeagus darker; inner gonostylus with two basal prolongations: dorsal prolongation (Figure 9D) T-shaped, upper arm bearing two curved black apical spines about half length of upper arm, lower arm with 1–3 setae at subtip; ventral prolongation long, pointing upward, with a apical spine, about 1/2 length of ventral prolongation. Paramere (Figure 9E–G) finger-shaped, broad at base, narrowed and slightly outwardly curved apically, with extreme tip bending inward slightly. Aedeagus (Figure 9E–G) long, extending to about middle of inner gonostylus, narrowed gradually and recurved, tip forked and bluntly rounded.

Female. Unknown.

Etymology. The specific name from Latin bispinosa (adj., meaning “bispinous”) refers to the upper arm of the T-shaped dorsal prolongation of the inner gonostylus bearing two curved black apical spines.

Distribution. China (Yunnan).

Remarks. This new species can be easily distinguished from other species of the subgenus by the characteristic of two spines at the upper arm of the dorsal prolongation of the inner gonostylus. Additionally, it can be further separated from the Chinese species *D*. (*S*.) *alticola* Edwards, 1916 and the newly described *D*. (*S*.) *inflata* sp. nov. by detailed hypopygial characteristics. In *D*. (*S*.) *alticola*, the posterior margin of tergite 9 has a broad and distinct U-shaped notch; the upper arm of the dorsal prolongation of the inner gonostylus is slender and subtip curved, with an apical spine. In *D*. (*S*.) *inflata*, the posterior margin of tergite 9 also has a broad and distinct U-shaped notch; the upper arm of the dorsal prolongation of the inner gonostylus is expanded, with an apical spine. However, in the new species, the posterior margin of tergite 9 is medially shallowly concave, bearing a median dark brown longitudinal line; the upper arm of the dorsal prolongation of the inner gonostylus has two curved black apical spines. In addition, this new species resembles *D*. (*S*.) *fortis* Brunetti, 1912 in the termination point of vein Sc, but can be distinguished by detailed head characters and body size. In *D*. (*S*.) *fortis*, the head has a very wide frons, about two-thirds the width of the head at the vertex, eyes contiguous below for a short space, and body length between 4.5 and 5.5 mm [7]. In this species, the head does not possess the above features, and the body length is 7.5 mm.

#### 3.2.3. *Dicranomyia* (*Sivalimnobia*) *inflata* Dai & Zhang, sp. nov.

ZooBank registration number of the new species: urn:lsid:zoobank.org:act:52B3DF35-D7D8-4141-8E03-6666C5460C66.

Type material. Holotype 1 male, China: Yunnan Province, Lushui County (2887 m), 10 July 2016, Liang Wang. Paratype: 1 female, China: Chongqing Municipality, Jiangjin District, Simian Mountain, Dahonghai (1165 m), 6 July 2018 (light trap). 1 female, China: Sichuan Province, Emeishan City, Mount Emei, Jinding, 7 July 2019. 1 female, 1 male, China: Sichuan Province, Pingwu County, Wanglang Nature Reserve, 30 July 2017, Yizhe Li (light trap). 1 male, China: Sichuan Province, Pingwu County, Wanglang Nature Reserve (2500 m), 6 July 2015, Yuqiang Xi. 1 male, China: Sichuan Province, Pingwu County, Wanglang Nature Reserve (2500 m), 8 August 2016, Yuqiang Xi. 2 females, China: Sichuan Province, Pingwu County, Wanglang Nature Reserve, Baihualin (2504 m), 23 July 2016, Yizhe Li (light trap). 1 male, China: Sichuan Province, Pingwu County, Wanglang Nature Reserve, Baishachang (3031 m), 20 July 2016, Yizhe Li (light trap). 1 male, China: Sichuan Province, Pingwu County, Wanglang Nature Reserve, Baixionggou (2864 m), 24 July 2016, Yizhe Li (light trap). 1 male, China: Sichuan Province, Pingwu County, Wanglang Nature Reserve, Dawodang (2914 m), 21 July 2016, Yizhe Li (light trap). 3 females, 1 male, China: Sichuan Province, Pingwu County, Wanglang Nature Reserve, Qikeshu (2446 m), 23 July 2016, Yizhe Li (light trap). 7 males, China: Sichuan Province, Pingwu County, Shuizhagou (2447 m), 20 July 2016, Yizhe Li (light trap). 1 female, 11 males, China: Sichuan Province, Yajiang County, Xianggezong Village (3579 m), 1 July 2019, Liang Wang. 1 male, China: Yunnan Province, Lushui County (2887 m), 10 July 2016, Liang Wang. 1 male, China: Yunnan Province, Lvchun County, Yakou Reservoir (1779 m), 16 March 2019, Liang Wang.

Diagnosis. Prescutum and presutural scutum varied from pale yellow to dark brown, with three stripes in darker individuals, median broad, laterals narrow. Postsutural scutum varied from pale yellow to brown, with a pale area medially when it is dark. Pleuron yellow, darker near base of wing. Femora uniformly pale yellow. Vein Sc ending from just beyond origin to 2/5 length of Rs; sc-r close to tip of Sc (up to 2.0 times its own length). Dorsal prolongation of inner gonostylus T-shaped, upper arm expanded, with an apical spine about half length of upper arm. Paramere finger-shaped, broad at base, tip acute, with subtip distinctly bent. Aedeagus slightly widened at base, narrowed gradually and recurved, tip forked and recurved, subtip distinctly constricted.

Description. Male. Body length 7.7–8.4 mm, wing length 8.0–9.0 mm.

**Figure 10:**
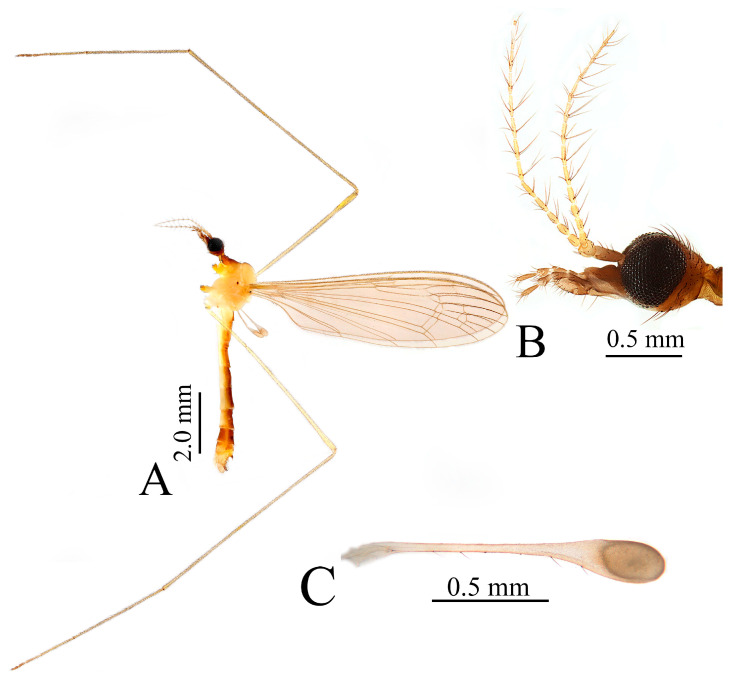
*Dicranomyia* (*Sivalimnobia*) *inflata* sp. nov.: (**A**) habitus of male, lateral view; (**B**) head, lateral view; (**C**) halter.

Head (Figure 10A,B). Brown. Setae on head brown. Antenna with 14 segments, brownish yellow with scape and pedicel darker. Scape cylindrical, 3.0 times as long as wide. Pedicel oval, length and width subequal. Basal three flagellomeres short-oval, remaining flagellomeres elongated, tapering apically; each flagellomere with 4–6 brown verticils, disposed in an irregular whorl, and longest exceeding length of corresponding flagellomere, outermost flagellomere with two short apical setae. Rostrum brown with dark brown setae. Palpus dark brown with dark brown setae.

**Figure 11:**
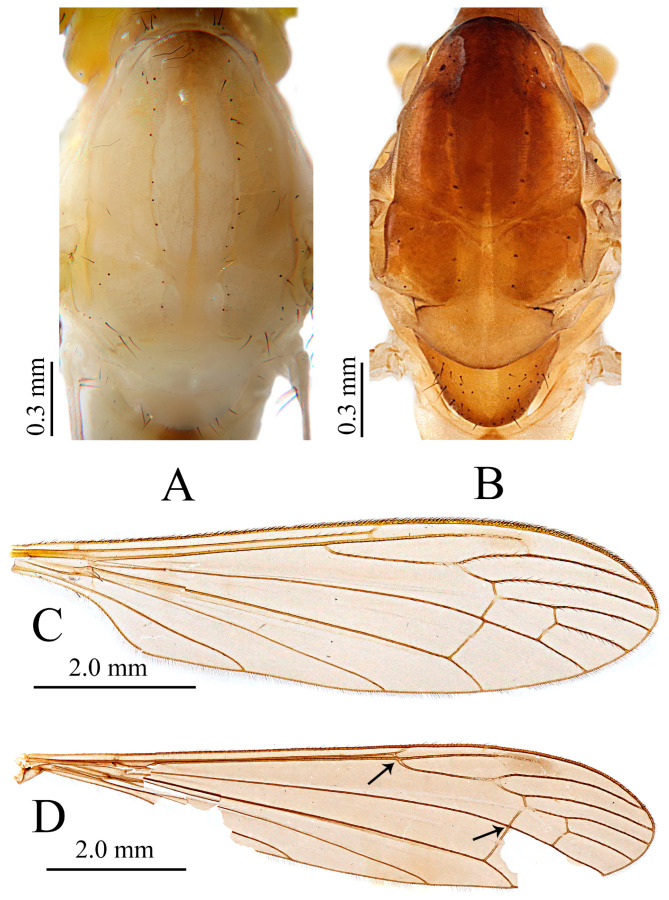
*Dicranomyia* (*Sivalimnobia*) *inflata* sp. nov.: (**A**,**B**) variations in thorax, dorsal view; (**C**,**D**) variations in wings. The black arrows in (**D**) indicate intraspecific variation in the termination point of Sc and sc-r, and in the position of m-cu; these features are not taxonomically diagnostic within *D*. (*S*.) *inflata*.

Thorax (Figure 10A and Figure 11A,B). Cervical sclerite dark brown. Pronotum brownish yellow, with anterior area darker. Prescutum and presutural scutum varied from pale yellow to dark brown (Figure 11A,B), with three stripes in darker individuals, median broad, laterals narrow (Figure 11B). Postsutural scutum varied from pale yellow to brown (Figure 11A,B), with a pale area medially when it is dark (Figure 11B). Scutellum varied from yellowish white to pale brown (Figure 11A, B). Mediotergite varies from yellowish white to pale brown (Figure 11A,B), with a darker area on posterior margin when it is dark (Figure 11B). Pleuron yellow, darker near base of wing (Figure 10A). Thorax with dark brown setae, densely covered with fine brown pubescence (Figure 11A,B). Coxae golden yellow (Figure 10A); trochanters slightly darker than coxae; femora uniformly pale yellow; tibiae brownish yellow; tarsi pale brown. Setae on legs brown. Wing brownish yellow to brown, stigma faint and slightly darker; veins brown; Venation: Sc ending from just beyond origin to 2/5 length of Rs (Figure 11C,D); sc-r close to tip of Sc (up to 2.0 times its own length); Rs arising from middle to just beyond middle of wing, base slightly curved; cell dm closed, 1.5–2.0 times as long as wide; m-cu at (Figure 11D) or just beyond (Figure 11C) base of dm. Halter (Figure 10C) brownish yellow, knob darker.

Abdomen (Figure 10A). Segments 1–6 with tergites brownish yellow and sternites pale yellow; segment 7 brown, segment 8 brownish yellow with anterior half darker. Setae on abdomen brown.

**Figure 12:**
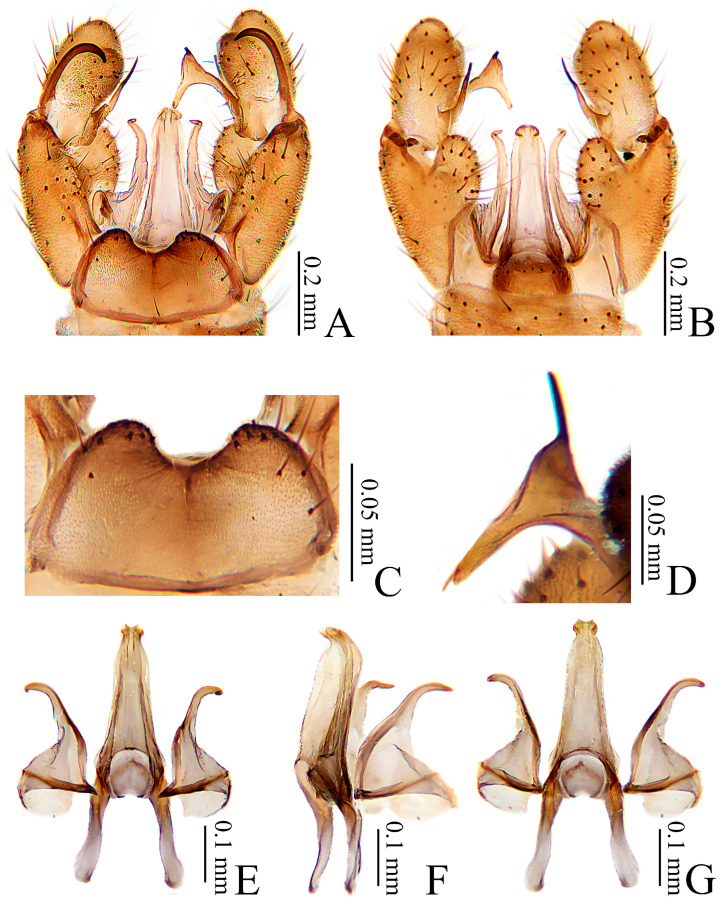
*Dicranomyia* (*Sivalimnobia*) *inflata* sp. nov.: (**A**) male hypopygium, dorsal view; (**B**) male hypopygium, ventral view; (**C**) tergite 9, dorsal view; (**D**) dorsal prolongation, dorsal view; (**E**) complex of aedeagus, dorsal view; (**F**) complex of aedeagus, lateral view; (**G**) complex of aedeagus, ventral view.

Hypopygium (Figure 12). Generally brown. Tergite 9 (Figure 12A,C) nearly trapeziform, posterior margin of tergite 9 with a broad and distinct U-shaped notch, each lobe with about 8–11 long dark brown setae. Gonocoxite (Figure 12A,B) cylindrical, slightly tapered at tip, with a moderate ventromesal lobe at middle, length about half of gonocoxite; gonocoxite with dark brown setae, ventromesal lobe with dense long brown setae. Outer gonostylus (Figure 12A,B) sclerotized, constricted basally, apical third incurved, brownish black with tip darker. Inner gonostylus (Figure 12A,D) slightly longer than outer gonostylus, shallowly concave at middle of upper margin, overall brown except for paler base laterally; inner gonostylus with two basal prolongations: dorsal prolongation (Figure 12D) T-shaped, upper arm expanded, with an apical spine about half length of upper arm; lower arm with 1–2 setae at subtip; ventral prolongation long, pointing upward, with a apical spine about 1/3 length of ventral prolongation. Paramere (Figure 12E–G) finger-shaped, broad at base, narrowed and slightly outward curved apically, tip acute, with subtip distinctly bent. Aedeagus (Figure 12E–G) moderately long, extending to about base of inner gonostylus, slightly widened at base, narrowed gradually and recurved, tip forked and recurved, subtip distinctly constricted.

**Figure 13:**
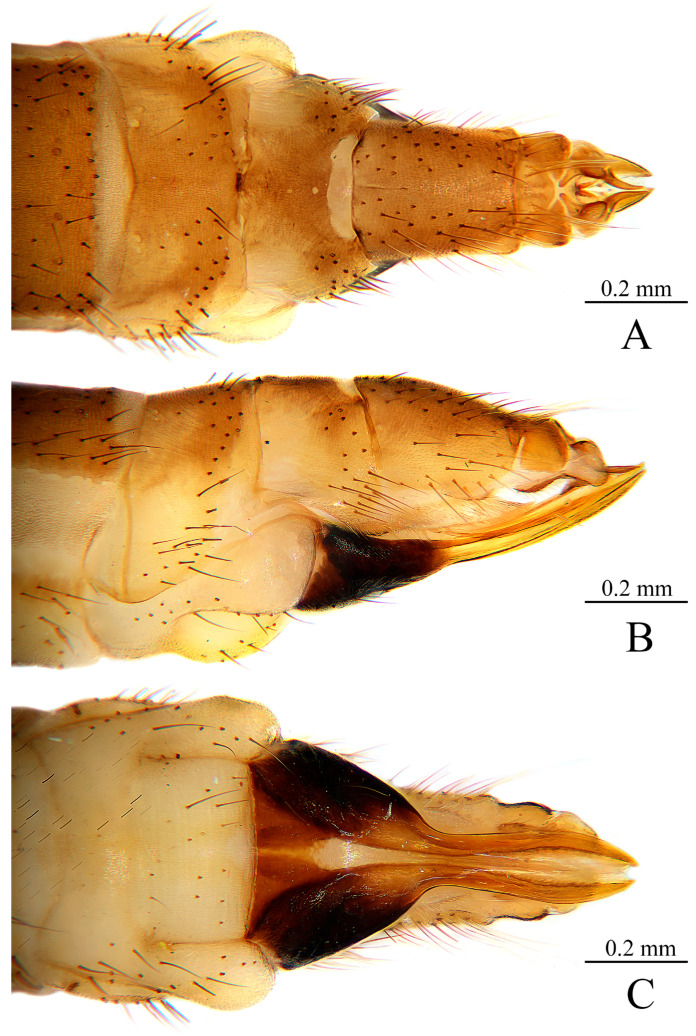
*Dicranomyia* (*Sivalimnobia*) *inflata* sp. nov.: (**A**) female ovipositor, dorsal view; (**B**) female ovipositor, lateral view; (**C**) female ovipositor, ventral view.

**Figure 14:**
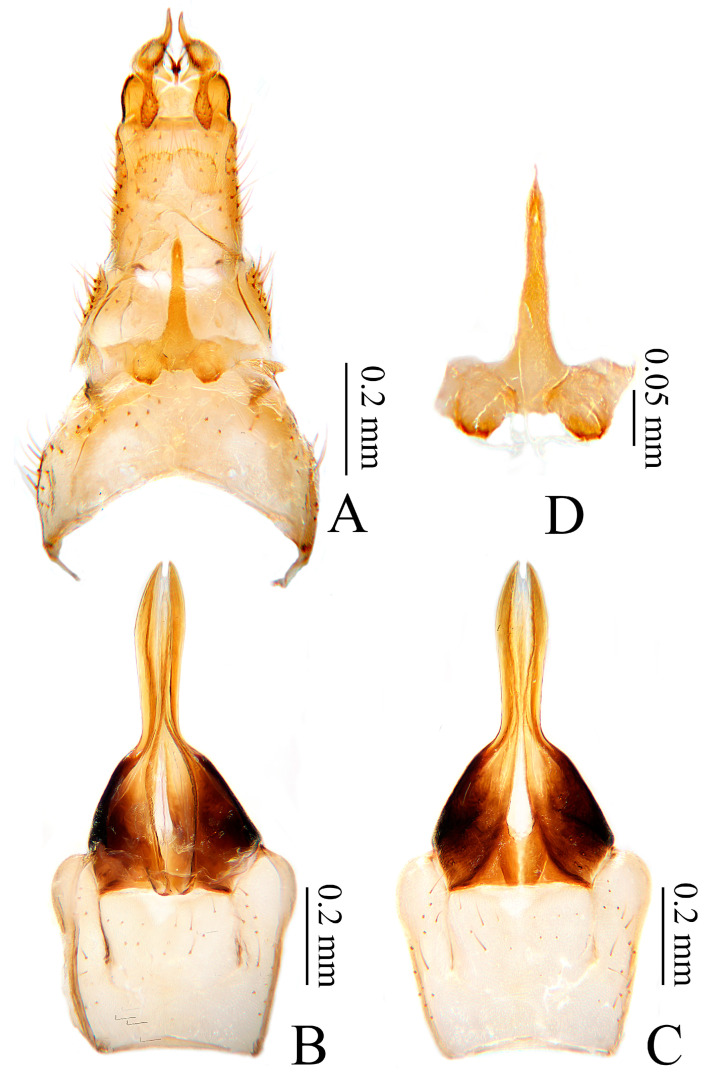
*Dicranomyia* (*Sivalimnobia*) *inflata* sp. nov.: (**A**) female ovipositor with sternite 8 and hypogynial valvae removed for clarity, ventral view; (**B**) sternite 8 with hypogynial valve, dorsal view; (**C**) sternite 8 with hypogynial valve, ventral view; (**D**) sternite 9, ventral view.

Female. Body length 7.2–8.0 mm, wing length 8.2–9.1 mm. Generally like male in body coloration. Ovipositor (Figure 13 and Figure 14) with tergite 8 about 2/3 as long as tergite 9, yellow, medial and caudal margin darker. Tergite 9 brownish yellow. Tergite 10 about 1/3 as long as tergite 9, brownish yellow, slightly convex in lateral view. Cercus short, apically acute, yellowish brown. Hypogynial valve pale brownish yellow and medially paler, with base brownish black and slightly swollen (visible in dorsal view). Sternite 9 (Figure 14A,D) triangular, with two subcircular lobes at base, apically tapering.

Etymology. The specific name from Latin inflata (adj., meaning “inflated”) refers to the upper arm of the T-shaped dorsal prolongation of the inner gonostylus, which is inflated.

Distribution. China (Chongqing, Sichuan, Yunnan).

Remarks. This new species can be distinguished from the known Chinese *D*. (*S*.) *alticola* Edwards, 1916 and the newly discovered *D*. (*S*.) *bispinosa* sp. nov. by specific details of the upper arm of the dorsal prolongation of the inner gonostylus. In *D*. (*S*.) *alticola*, the upper arm of the dorsal prolongation of the inner gonostylus bears a single spine and is curved at the base of the spine; in *D*. (*S*.) *bispinosa* sp. nov., the upper arm of the dorsal prolongation of the inner gonostylus bears two curved spines, whereas in this new species, the upper arm of the dorsal prolongation of the inner gonostylus is simply expanded. In addition, this new species can be further separated from *D*. (*S*.) *alticola* by the color of the femora: in *D*. (*S*.) *alticola*, each femur has a broad dark apical ring, whereas in this new species, the femora are entirely yellow. Although all three Chinese species have a constricted subapical portion of the aedeagus, this constriction is more distinct in this species.

While this new species is similar to *D*. (*S*.) *rahula* (Alexander, 1963), described by Alexander from Uttarakhand, India in 1963, in hypopygial characteristics, it can still be distinguished by the degree of swelling of the upper arm of the dorsal prolongation of the inner gonostylus and the coloration of the femora. In *D*. (*S*.) *rahula*, the upper arm of the dorsal prolongation of the inner gonostylus is swollen and triangular in shape, and the femora are yellow with the tips very narrowly brown [2], whereas in this new species, the upper arm of the dorsal prolongation of the inner gonostylus is more strongly swollen and arcuate in shape, and the femora are entirely yellow. This new species also resembles *D*. (*S*.) *pererratica* (Alexander, 1973) in head morphology and overall body length and size but can be distinguished by details of the male hypopygium. In *D*. (*S*.) *pererratica*, the outer gonostylus is lacking, the lower arm of the dorsal prolongation of the inner gonostylus bears a conspicuous sclerotized flange with five small marginal points, and the paramere tip is obtuse [17], whereas in this species, the outer gonostylus is present, the lower arm of the dorsal prolongation of the inner gonostylus is unmodified, bearing only 1–2 setae subapically, and the paramere tip is acute.

#### 3.2.4. Key to Chinese Species of *Sivalimnobia*

1. Posterior margin of tergite 9 medially shallowly concave, bearing a median dark brown longitudinal line (Figure 9C); dorsal prolongation of inner gonostylus with two curved black apical spines on upper arm (Figure 9D)................................................................................................*Dicranomyia* (*Sivalimnobia*) *bispinosa* Dai & Zhang, sp. nov.
  Posterior margin of tergite 9 with a broad and distinct U-shaped notch; dorsal prolongation of inner gonostylus with one apical spine on upper arm................................2
2. Femora brownish yellow with a broad dark apical ring (Figure 2A); upper arm of dorsal prolongation of inner gonostylus slender and curved at subtip (Figure 4D); paramere tip acute with extreme tip bending inward abruptly (Figure 4E–G).............................................................................*Dicranomyia* (*Sivalimnobia*) *alticola* Edwards, 1916
  Femora uniformly pale yellow (Figure 10A); upper arm of dorsal prolongation of inner gonostylus expanded (Figure 12D); paramere tip acute with subtip distinctly bending (Figure 12E–G)...........*Dicranomyia* (*Sivalimnobia*) *inflata* Dai & Zhang, sp. nov.

### 3.3. Geographic Distribution

**Figure 15:**
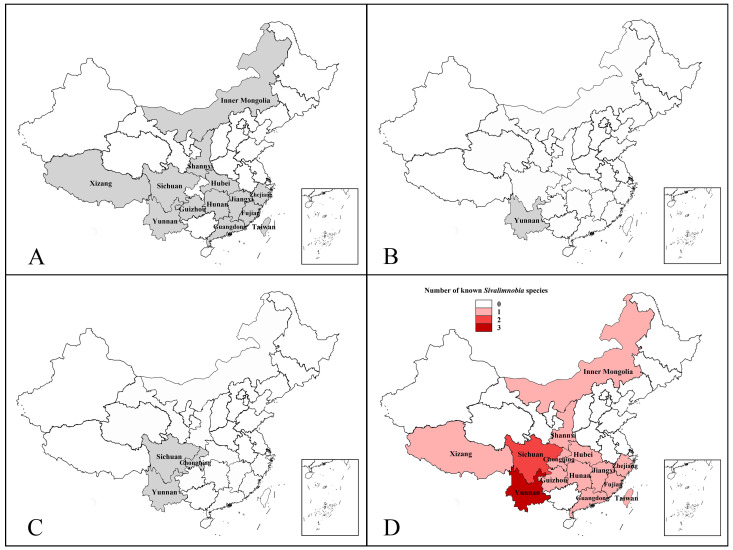
Distribution maps of *Sivalimnobia* in China: (**A**) *Dicranomyia* (*Sivalimnobia*) *alticola* Edwards, 1916; (**B**) *D*. (*S*.) *bispinosa* sp. nov.; (**C**) *D*. (*S*.) *inflata* sp. nov.; (**D**) species richness pattern across the range of the subgenus.

This study establishes the first confirmed presence of the subgenus *Sivalimnobia* in mainland China, addressing a major biogeographic knowledge gap. The specimens were collected from 14 provincial-level regions, confirming three distinct species within the subgenus. The most significant finding is the remarkable range expansion of *D*. (*S*.) *alticola*, which was previously known only from Taiwan (China) and Bali (Indonesia). We documented this species across 13 provincial-level regions (Figure 15A), from Yunnan in the southwest (∼22° N) to Inner Mongolia in the north (∼39° N), representing a latitudinal extension of approximately 18 degrees. This transforms its status from an island-restricted taxon to a continentally distributed species. Concurrent with the range expansion of a known species, we discovered two narrow-range endemic species confined to southwestern China. *D*. (*S*.) *inflata* sp. nov. is distributed along the eastern margin of the Hengduan Mountains in the contiguous area of Sichuan, Yunnan and Chongqing (Figure 15C), while *D*. (*S*.) *bispinosa* sp. nov. is currently known only from a limited area in Yunnan (Figure 15B). Both species exhibit point or linear distribution patterns associated with specific montane habitats, contrasting sharply with the broad distribution of *D*. (*S*.) *alticola*.

Geographically, the subgenus in China exhibits a clear center–periphery pattern. The Hengduan Mountains region spanning Yunnan and Sichuan serves as the diversity center, hosting all three species (Figure 15D). Within this core area, species richness decreases from Yunnan (three species) to Sichuan (two species), reflecting the topographical complexity and habitat heterogeneity of this montane region. This richness gradient underscores the role of the Hengduan Mountains as both a diversification hub and a filter for eastward dispersal. This area represents both a glacial refugium for the two narrow-range endemics and a likely source for recent eastward range expansion of *D*. (*S*.) *alticola*. From this center, the widespread *D*. (*S*.) *alticola* has expanded across 11 other provincial-level regions across China, ranging from the southwest to the north and east and extending to Taiwan, demonstrating its ecological adaptability to diverse climatic zones from subtropical to temperate regions.

Integrating our findings with historical records reveals a revised hierarchical structure for the subgenus (Table 3). India is a primary diversity center with nine species. However, southwestern China now emerges as a secondary diversity center with three species (including two endemics). The subgenus displays three distinct dispersal frontiers: the European temperate frontier (*D*. (*S*.) *aquosa*, continuous distribution), the East Asian-island frontier (e.g., *D*. (*S*.) *euphileta*, disjunct distribution), and now the continental East Asian frontier (*D*. (*S*.) *alticola*, broad continuous distribution). We think a “stepping-stone dispersal and multi-center diversification” hypothesis may be invoked to explain these patterns. The Sino-Himalayan region served as both a diversification hotspot (generating *D*. (*S*.) *bispinosa* sp. nov. and *D*. (*S*.) *inflata* sp. nov.) and a critical biogeographic gateway for eastward dispersal. The ecological versatility demonstrated by *D*. (*S*.) *alticola* in colonizing diverse habitats, from subtropical to temperate zones, provides the adaptive basis for such widespread distribution.

## 4. Discussion

This integrative study updates our understanding of the dual enigmas of taxonomic stasis and biogeographic anomaly in *Sivalimnobia*. The discovery of three species in China—comprising two new endemics and one dramatically range-expanded species—further reshapes understanding of the subgenus. The cryptic divergence within the *D*. (*S*.) *alticola* complex is consistently supported by both concordant morphological and molecular evidence, validating the precision of male genitalic characters for species delimitation in this group. COI barcode distances between the two clades (8.7–9.6%) fall within the range reported for *Limonia* (7.9–17.2%) [28] and *Discobola* (7.6–17.7%) [29], while intraspecific distances (0–2.2%) remain low, consistent with patterns observed in these related genera. This congruence between independent datasets demonstrates the critical role of integrative taxonomy in overcoming historical reliance on variable traits.

Our findings further reveal the biogeographic template of *Sivalimnobia*, with an Indian diversity core, a newly identified Sino-Himalayan secondary center, and three distinct distributional frontiers. This pattern is suggestive of a “stepping-stone diversification” hypothesis. This modern biogeographic structure may be further contextualized by a fossil representative of *Sivalimnobia* documented from Eocene Baltic amber [30]; the observed Sino-Indian diversity centers may represent the most recent expression of a much longer evolutionary history. The widespread *D*. (*S*.) *alticola* exemplifies an ecologically versatile species that has achieved a broad continental distribution, while the two new point-endemic species represent paleoendemics specialized to montane refugial habitats within the Hengduan Mountains.

This research carries immediate implications for conservation and future study. The micro-endemic new species are inherently vulnerable and highlight the urgent need to protect southwestern China’s montane stream ecosystems. The expanded range of *D*. (*S*.) *alticola* provides a model system for studying adaptive dispersal, while the revised species boundaries establish an essential foundation for future phylogenetic and phylogeographic work.

## 5. Conclusions

This study breaks a five-decade period of taxonomic stasis in *Sivalimnobia* through an integrative approach, conclusively establishing its presence in mainland China with the discovery of three species: the newly recorded and continentally widespread *D*. (*S*.) *alticola*, along with two endemic new species from southwestern China. Our methodology resolved a cryptic lineage within the *D*. (*S*.) *alticola* complex by integrating congruent morphological and molecular evidence. The emergent biogeographic pattern—featuring India as the primary diversity center and southwestern China as a newly identified secondary center—suggests a “stepping-stone dispersal” model. Collectively, these findings elevate *Sivalimnobia* from a taxonomically stagnant group to a compelling model for Eurasian biogeographic study, while underscoring the conservation significance of specialized montane riparian habitats for insect diversity.

## Figures and Tables

**Table 1:** Information of *Sivalimnobia* specimens sequenced in this study.

Sample ID	Species	Sex	Locality	Process ID
DSIAL01	*D*. (*S.*) *alticola*	male	CHINA: Hunan Province, Zhangjiajie City, Sangzhi County, Tianping Mountain (1300 m)	DBDSC001-26
DSIAL02	*D*. (*S*.) *alticola*	male	CHINA: Zhejiang Province, Suichang County, Jiulong Mountain, Xikengli Protection Station	DBDSC002-26
DSIAL03	*D*. (*S*.) *alticola*	male	CHINA: Yunnan Province, Lvchun County, Yakou Reservoir (1779 m)	DBDSC003-26
DSIAL04	*D*. (*S*.) *inflata*	male	CHINA: Sichuan Province, Yajiang County, Xianggezong Village (3579 m)	DBDSC004-26
DSIAL05	*D*. (*S*.) *inflata*	female	CHINA: Sichuan Province, Pingwu County, Wanglang Nature Reserve	DBDSC005-26
DSIAL06	*D*. (*S*.) *inflata*	male	CHINA: Yunnan Province, Lushui County (2887 m)	DBDSC006-26

**Table 2:** Genetic distance matrix showing the divergence between *Dicranomyia* (*Sivalimnobia*) mt COI sequences.

Species (Process ID)	*D.* (*S.*) *alticola* (DBDSC001-26)	*D.* (*S.*) *alticola* (DBDSC002-26)	*D.* (*S.*) *alticola* (DBDSC003-26)	*D.* (*S.*) *inflata* (DBDSC004-26)	*D.* (*S.*) *inflata* (DBDSC005-26)	*D.* (*S.*) *inflata* (DBDSC006-26)
***D.*** **(*S.*)** ***alticola*** **(DBDSC001-26)**						
***D.*** **(*S.*)** ***alticola*** **(DBDSC002-26)**	0.2% *					
***D.*** **(*S.*)** ***alticola*** **(DBDSC003-26)**	0.2% *	0.0% *				
***D.*** **(*S.*)** ***inflata*** **(DBDSC004-26)**	9.0% ^#^	8.7% ^#^	8.7% ^#^			
***D.*** **(*S.*) *inflata* (DBDSC005-26)**	9.6% ^#^	9.6% ^#^	9.6% ^#^	1.9% *		
***D.*** **(*S.*)** ***inflata*** **(DBDSC006-26)**	9.0% ^#^	9.0% ^#^	9.0% ^#^	1.5% *	2.2% *	

* Intraspecific distances. ^#^ Interspecific distances.

**Table 3:** World distribution of the subgenus *Sivalimnobia* species.

Taxon Name	Distribution
**Indian region** *	
*D*. (*S*.) *approximata* Brunetti, 1912	India: West Bengal
*D*. (*S*.) *bicolor* Brunetti, 1918	India: Assam
*D*. (*S*.) *clavula* (Alexander, 1972)	India: Assam
*D*. (*S*.) *fortis* Brunetti, 1912	India: West Bengal
*D*. (*S*.) *kali* (Alexander, 1963)	India: West Bengal
*D*. (*S*.) *marginella* (Alexander, 1929)	India: Assam
*D*. (*S*.) *pererratica* (Alexander, 1973)	India: Assam
*D*. (*S*.) *rahula* (Alexander, 1963)	India: Uttarakhand
*D*. (*S*.) *uma* (Alexander, 1967)	India: Assam
**Southwestern China** region *	
*D*. (*S*.) *alticola* Edwards, 1916	China: Fujian, Guangdong, Guizhou, Hubei, Hunan, Inner Mongolia, Jiangxi, Shaanxi, Sichuan, Taiwan, Xizang, Yunnan, Zhejiang; Indonesia: Bali
*D*. (*S*.) *bispinosa* sp. nov.	China: Yunnan
*D*. (*S*.) *inflata* sp. nov.	China: Chongqing, Sichuan, Yunnan
**European temperate lineage** *	
*D*. (*S*.) *aquosa* (Verrall, 1886)	Albania; Andorra; Austria; Belgium; Bulgaria; France (incl. Corsica); Germany; Great Britain; Ireland; Italy; Romania; Slovakia; Slovenia; Spain; Switzerland
**East Asian island-arc lineage** *	
*D*. (*S*.) *euphileta* (Alexander, 1924)	Japan: Honshu, Shikoku, Kyushu; Malaysia: Sabah (Borneo); Russia: Sakhalin, Kuril Islands; South Korea
*D*. (*S*.) *excelsa* Alexander, 1915	Indonesia: Java
*D*. (*S*.) *nongkodjadjarensis* (Meijere, 1913)	Indonesia: Java, Bali; Malaysia: Peninsular Malaysia, Sabah (Borneo)
*D*. (*S*.) *pleiades* (Alexander, 1931)	Philippines: Mindanao

* Bold headings denote geographic regions or lineage groups, as delimited based on literature records and the present findings.

## Data Availability

The mitochondrial COI sequences obtained in this study are publicly available in the BOLD database (http://www.boldsystems.org/, accessed on 26 February 2026) under accession numbers DBDSC001-26 to DBDSC006-26, where only the numerical portion preceding the hyphen increases sequentially.
